# Bidirectional Regulation of Hippocampal Synaptic Plasticity and Modulation of Cumulative Spatial Memory by Dopamine D2-Like Receptors

**DOI:** 10.3389/fnbeh.2021.803574

**Published:** 2022-01-12

**Authors:** Violeta-Maria Caragea, Denise Manahan-Vaughan

**Affiliations:** ^1^Department of Neurophysiology, Medical Faculty, Ruhr University Bochum, Bochum, Germany; ^2^International Graduate School of Neuroscience, Ruhr University Bochum, Bochum, Germany

**Keywords:** hippocampus, synaptic plasticity, long-term potentiation, spatial memory, dopamine D2 receptors (DRD2), episodic, semantic, rodent

## Abstract

Dopamine is a key factor in the enablement of cognition and hippocampal information processing. Its action in the hippocampus is mediated by D1/D5 and D2-like (D2, D3, D4) receptors. While D1/D5-receptors are well recognized as strong modulators of hippocampal synaptic plasticity and information storage, much less is known about the role of D2-like receptors (D2R) in these processes. Here, we explored to what extent D2R contribute to synaptic plasticity and cumulative spatial memory derived from semantic and episodic-like information storage. In freely behaving adult rats, we also assessed to what extent short and long-term forms of synaptic plasticity are influenced by pharmacological activation or blockade of D2R. Antagonism of D2R by means of intracerebral treatment with remoxipride, completely prevented the expression of both short-term (<1 h) and long-term potentiation (>4 h), as well as the expression of short-term depression (STD, <1 h) in the hippocampal CA1 region. Scrutiny of involvement of D2R in spatial learning revealed that D2R-antagonism prevented retention of a semantic spatial memory task, and also significantly impaired retention of recent spatiotemporal aspects of an episodic-like memory task. Taken together, these findings indicate that D2R are required for bidirectional synaptic plasticity in the hippocampal CA1 region. Furthermore, they are critically involved in enabling cumulative and episodic-like forms of spatial learning.

## Introduction

The hippocampus is a key structure for experience-dependent information storage and memory formation (Manns and Eichenbaum, 2006). Research findings of the last decades support that synaptic plasticity in the hippocampus is an essential mechanism underlying experience-dependent learning and memory processes (Manahan-Vaughan and Braunewell, 1999; Kemp and Manahan-Vaughan, 2004; Gruart et al., 2006; Whitlock et al., 2006; Goh and Manahan-Vaughan, 2013). Both long-term potentiation (LTP) and long-term depression (LTD) are modulated by dopamine (DA; Jay, 2003; Sajikumar and Frey, 2004; Lemon and Manahan-Vaughan, 2006; Bäckman et al., 2010; Bethus et al., 2010; Ortiz et al., 2010; Hansen and Manahan-Vaughan, 2014; Kempadoo et al., 2016; Edelmann and Lessmann, 2018). For example, DA contributes to the fine-tuning of hippocampal memory acquisition (Heath et al., 2015) and consolidation (Sara et al., 1999; Atherton et al., 2015). It has been proposed that the DA tonus acts as a novelty signal that drives the induction and maintenance of hippocampal synaptic plasticity (Lisman and Grace, 2005; Hansen and Manahan-Vaughan, 2014).

In the hippocampus, DA acts on two subgroups of receptors: the D1-like receptors (D1 and D5) that are positively coupled to adenylyl cyclase (AC) and the D2-like receptors (D2R; including D2, D3, and D4 receptors) that are negatively coupled to AC (Beaulieu and Gainetdinov, 2011). The role of D1-like receptors in regulating the duration and stability of synaptic plasticity at diverse synaptic populations within the hippocampal trisynaptic circuit has been closely studied (Hansen and Manahan-Vaughan, 2014). Activation of these receptors is important for the maintenance and longevity of long-term potentiation (LTP) *in vivo* and *in vitro* (Sajikumar and Frey, 2004; Swant and Wagner, 2006; Navakkode et al., 2007; Granado et al., 2008; Hagena and Manahan-Vaughan, 2016; Twarkowski and Manahan-Vaughan, 2016; Guo et al., 2017; Papaleonidopoulos et al., 2018), while their antagonism curtails the duration of LTP, LTD, and depotentiation evoked in both the hippocampal dentate gyrus and cornus ammonis (CA) region of freely behaving adult rats (Swanson-Park et al., 1999; Lemon and Manahan-Vaughan, 2006; Granado et al., 2008; Wiescholleck and Manahan-Vaughan, 2014; Broussard et al., 2016; Hagena and Manahan-Vaughan, 2016; Navakkode et al., 2017). By contrast, the involvement of D2R in synaptic plasticity in the CA1 region of behaving rats has been subjected to less scrutiny. However, it has been reported that these receptors are not required for mossy fiber LTP or LTD in the CA3 region *in vivo* (Hagena and Manahan-Vaughan, 2016), whereas in the dentate gyrus (DG), receptor antagonism prevents weak but not strong forms of LTP in behaving rats (Manahan-Vaughan and Kulla, 2003). Mice lacking D2R also show reduced LTP (Espadas et al., 2021). D2R activation in the hippocampus has been reported to lower excitability levels and, thus, raises the threshold for induction of LTP (Manahan-Vaughan and Kulla, 2003).

To what extent synaptic plasticity in the hippocampal CA1 region *in vivo* is affected by D2R is as yet unclear. Nonetheless, their presence in the CA1 region was already clearly demonstrated (Charuchinda et al., 1987; Dubovyk and Manahan-Vaughan, 2019; Yu et al., 2019). *In vitro* studies, reported rather contradictory findings, however. For example, in an older study, it was shown that various D2R antagonists had no effect on the induction of LTP, although they gradually and significantly diminished its maintenance (Frey et al., 1990). A more recent study reported that timing-dependent LTP was completely inhibited by D2 antagonism (Cepeda-Prado et al., 2021). *In vivo*, D2 antagonism inhibits kindling that is elicited by low frequency stimulation (LFS; Sadeghian et al., 2020). Moreover, studies in mice that either completely lack the D2 receptor, or where the receptor is silenced in the CA1 region *via* an siRNA approach, reported a significant reduction in LTP and synaptic depression (Rocchetti et al., 2015; Espadas et al., 2021). These findings suggest that D2R may be involved in regulating the bidirectionality of hippocampal synaptic plasticity: an aspect that we set about to clarify in the present study.

Behavioral studies of the role of D2R in different forms of memory have generated diverse results. On one hand, D2R-antagonism neither affects extinction learning of spatial appetitive experience (André and Manahan-Vaughan, 2016), nor appetitive reference memory (Daba Feyissa et al., 2019). On the other hand, the absence, or the inactivation, of D2R impairs trace eyeblink conditioning (Espadas et al., 2021) and spatial working memory (Glickstein et al., 2002; Rocchetti et al., 2015), as well as conditioned avoidance responses, passive avoidance retrieval and object recognition (Braszko, 2006; Prokopova et al., 2012). By contrast, receptor antagonism, when implemented post-encoding in a maze task, enhances memory (Sara, 1986; Setlow and McGaugh, 2000). Thus, the contribution of D2R to learning and memory may depend on the task, the treatment conditions, or even the dose of ligand used (Arnsten et al., 1995).

In the present study, we aimed to resolve these confounds by treating our animals with the same dose of antagonist used in our synaptic plasticity studies (using the same application route and treatment timing) and by assessing the effect of D2R antagonists on two facets of spatial memory, namely semantic and episodic-like object-place recognition memory. We report that D2R-antagonism prevents both synaptic potentiation and synaptic depression in the hippocampal CA1 region of freely behaving rats. Moreover, receptor antagonism significantly impairs retention of a semantic spatial memory task and also prevents memory of temporally proximal spatiotemporal aspects of an episodic-like task. Taken together, our data support a role for D2R in CA1 synaptic plasticity and in cumulative and episodic-like spatial memory in rodents.

## Materials and Methods

All experimental procedures were carried out in accordance with the guidelines of the European Communities Council Directive of September 22nd, 2010 (2010/63/EU) for the care of laboratory animals and after approval of the ethics committee of the federal state of North Rhine Westphalia (NRW; Landesamt für Naturschutz, Umweltschultz und Verbraucherschutz, NRW, Bezirksamt Arnsberg).

### Animals

Long Evans male rats (RGD Cat# 68073, RRID:RGD_68073, Charles River, Germany) were bred in-house. Experimental procedures started when an animal was at least 7–8 weeks old. Animals received *ad libitum* access to water and food. The rats were housed in ventilated cabinets (Scantainer, Scanbur Technology A/S, Denmark), kept at constant temperature (22 ± 2°C) and humidity (55 ± 5%) on a 12-h light-dark cycle (lights on at 7 a.m.). All tests were performed during the light cycle.

Different animal cohorts were used for the two behavioral studies. Some of the animals used for behavioral studies were also used in electrophysiological experiments. This was done to keep the animal numbers to a minimum. In this case, at least 7 days elapsed between the conclusion of an electrophysiological study and the commencement of a behavioral study, or *vice versa*. Animals were group-housed (3–4 animals/cage) prior to surgery and single-housed in translucent boxes with elevated mesh lids after surgery. The boxes were placed near one another in the housing cabinets so that the animals could see, sniff and hear one another.

### Surgery

Using stereotaxic coordinates, the animals were chronically implanted with hippocampal electrodes, and a guide cannula, under sodium pentobarbital anesthesia (52 mg/kg i.p., intraperitoneally). The implanted electrodes had a 0.1 mm diameter and were built from polyurethane-coated stainless-steel wires (Biomedical Instruments, Zöllnitz, Germany). The animals were implanted unilaterally in the right hemisphere with one monopolar recording electrode in the stratum radiatum of the dorsal hippocampal CA1 region (coordinates: −2.8 mm anterior from bregma; +1.8 mm from midline) and one bipolar stimulating electrode in the Schaffer collateral fibers (coordinates: −3.1 mm anterior from bregma; +3.1 mm from midline), as previously described (Manahan-Vaughan and Reymann, 1995b). The dorsoventral position (depth) was manually determined from the dura, based on the shape of the potential generated by the test-pulses input (Manahan-Vaughan, 2019). The guide cannula was implanted in the ipsilateral hemisphere (coordinates: −0.5 mm anterior from bregma; +1.6 mm from midline) to enable injections into the intracerebral ventricle (icv; Manahan-Vaughan, 1997). Grounding and reference wires were fixed to the bone *via* screws. After the electrodes were placed, the bone holes were sealed with surgical glue and a socket was built out of dental acrylic. Before, and after surgery (at 24 and 48 h), animals were treated subcutaneously with the analgesic Meloxicam (0.2 mg/kg; Metacam, Boehringer Ingelheim Vetmedica GmbH, Ingelheim/Rhein, Germany).

### Compounds

The D2R antagonist (S)- (–) -3- bromo- N-[(1-ethyl-2-pyrrolidinyl) methyl]-2,6 dimethoxybenzamide(remoxipride; Tocris, Bio-Techne GmbH, Germany) was dissolved in physiological saline solution (0.9% NaCl) to obtain a dose of 10 μg/μl, whereby animals were treated with 50 μg of the ligand intracerebrally. This dose was previously shown to modulate hippocampal LTP in the dentate gyrus of freely behaving rats, without affecting basal synaptic transmission (Manahan-Vaughan and Kulla, 2003). A total volume of 5 μl was administered in a single experiment, delivered gradually over a 5-min period by means of a Hamilton syringe (Hamilton Company, Reno, NV, USA) that was connected *via* tubing to a guide cannula. To enable the diffusion of the drug from the ventricle to the hippocampal region, the injection was carried out 30 min prior to applying any plasticity-inducing protocol, or to beginning a behavioral experiment (i.e., 30 min before Trial 1 was commenced).

### *In vivo* Electrophysiology

For every animal, a minimum period of 10 days was allocated for recovery before starting the first experiments. Animals were handled for at least 3 days before the first experiment, and handling was begun no earlier than 7 days after the surgery. One day before the experiment, the rats were placed in the recording room to acclimatize and habituate within the environment. The experiments were performed in 40 × 40 × 50 cm recording chambers with solid-gray washable acrylic walls and a transparent acrylic door, where animals had *ad libitum* access to food and water. Flexible cables, connecting a swivel with the animal’s socket, allowed the animals to move freely while evoked potentials were measured.

In order to evoke field excitatory postsynaptic potentials (fEPSPs), the Stratum radiatum of the CA1 region was stimulated *via* the Schaffer collaterals (SC) at a low frequency (0.025 Hz), with a single biphasic square waveform pulse (0.2 ms per half wave). First, a stimulus-response relationship was determined by applying test-pulses in steps of 100 μA, using stimulus intensities in the range of 100–900 μA. The stimulus intensity used to evoke potentials during the subsequent experiments was the value that induced an fEPSP that was 40% of the maximum slope obtained from the stimulus-response relationship. This was typically in the range of 50–150 μA.

**Figure 1:**
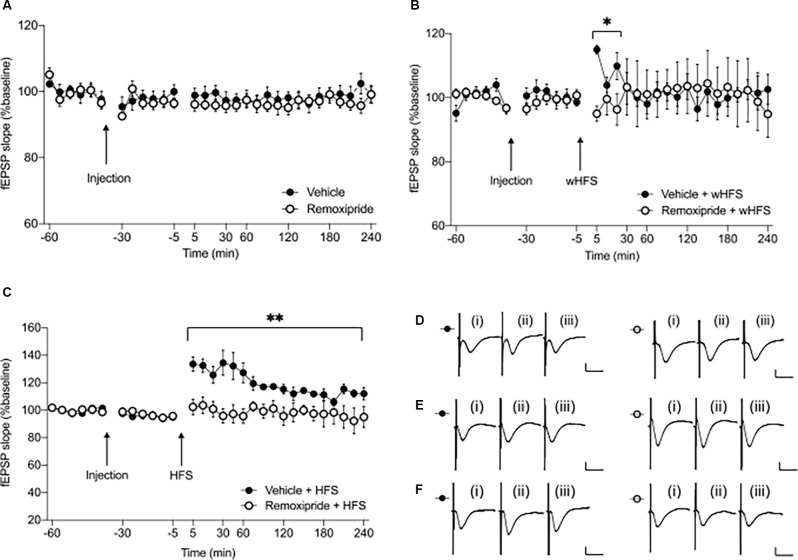
Pharmacological antagonism of D2-like receptors (D2R) prevents initiation of short and long-term synaptic potentiation at CA3-CA1 synapses. **(A)** The dopamine D2R antagonist remoxipride (50 μg, icv) does not alter basal synaptic transmission in freely behaving rats (*n* = 7, *p* = 0.40). **(B)** Application of weak high frequency stimulation (wHFS), in the presence of the D2-like receptor antagonist, remoxipride, prevents STP (*n* = 7, *p < 0.05). **(C)** Similarly, high frequency stimulation (HFS) in the presence of remoxipride inhibits LTP (*n* = 6, **p < 0.01). Arrows indicate time-points of injection or wHFS/HFS stimulation. Filled circles represent control experiments conducted in vehicle treated animals, while open circles indicate experiments where drug was applied prior to stimulation. Values represent ± SEM. **(D–F)** Analog responses recorded during remoxipride baseline experiments **(D)**, wHFS **(E)** or high frequency stimulation experiments **(F)**. In each case (i) shows an fEPSP evoked in the first 30 min of recordings; (ii) shows an fEPSP evoked at the 5 min time-point; and (iii) represents an fEPSP recorded at the 240 min timepoint; filled circles—vehicle treatment, open circles—remoxipride injection. Scale bars represent: 1 mV vertical/10 ms horizontal.

At least 7 days prior to starting synaptic plasticity experiments, a “baseline” experiment was conducted to check for accuracy and stability of basal synaptic transmission over the course of the experiment’s duration (ca. 4.5 h; Figure 1A). Evoked potentials were obtained by stimulating at low frequency (0.025 Hz) using single biphasic square-wave pulses of 0.2 ms duration. Each time-point consisted of five such stimuli. Five time-points were obtained over a 30 min period, then a vehicle injection was applied to the lateral ventricle. After a further 45 min of recordings at 5 min intervals, time-points were recorded at 15 min intervals until the experiment concluded, at a total of 5 h after commencing the experiment. Only animals that showed stable responses (100 ± 5%, calculated from the average of the first six baseline time-points) for the duration of the entire “baseline” experiment were included in subsequent experiments.

In order to induce synaptic potentiation, high frequency stimulation (HFS, 100 Hz) was used (Manahan-Vaughan and Reymann, 1995a; Manahan-Vaughan et al., 1999; Kemp and Manahan-Vaughan, 2004). To induce short-term potentiation (STP; <1 h), three bursts of 10 pulses only (one pulse lasting 0.1 ms and with 10 s inter-burst interval), were delivered (wHFS; see e.g., Figure 1B; filled circles). To obtain LTP (>4 h), HFS was delivered in four bursts, with a 5 min inter-burst interval, where each burst consisted of 30 pulses each (one pulse lasting 0.2 ms; see e.g., Figure 1C; filled circles). For both HFS protocols, the stimulus intensity was the same as for the test-pulses.

**Figure 2:**
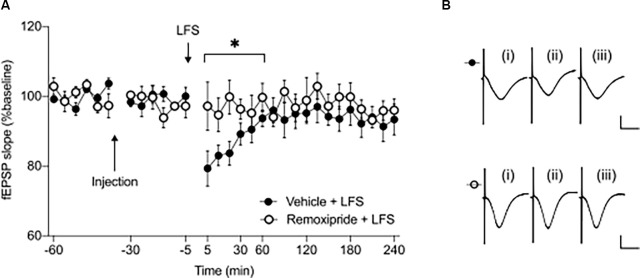
Short-term depression at the CA3-CA1 synapse is prevented by pharmacological antagonism of D2-like receptors (D2R). **(A)** Remoxipride (50 μg) blocked the initiation of short-term depression induced by low frequency stimulation (*n* = 6, ^*^p < 0.05). Arrows indicate the time-points of injection or low frequency stimulation. Filled circles represent the control experiments results, while open circles indicate experiments where drug was applied prior to LFS. Values are expressed as ± SEM. **(B)** Examples of analog responses recorded during low-frequency stimulation experiments with either vehicle treatment (filled circles) or remoxipride injection (open circles). In each case (i) shows an fEPSP evoked in the first 30 min of recordings; (ii) shows an fEPSP evoked at the 5 min time-point; and (iii) represents an fEPSP recorded at the 240 min timepoint. Scale bars represent: 1 mV vertical/10 ms horizontal.

To induce synaptic depression (<1 h), a low frequency stimulation (LFS) protocol was used (Strauch and Manahan-Vaughan, 2020), consisting of 1,800 paired-pulses (25 ms between the paired pulses) delivered at an intensity that elicited an fEPSP equivalent to 70% of the maximum one obtained in the stimulus-response relationship (Figure 2, filled circles). HFS, wHFS, or LFS, were applied 30 min after vehicle or ligand treatment, and a total of 60 min after the experiment was begun (see baseline experiment description above). After wHFS, HFS, or LFS, potentials were recorded for 4 h. Animals served as their own controls. This meant that the integrity of a synaptic plasticity event was verified by comparing evoked responses (for the duration of the experiment) with responses evoked in test-pulse stimulated controls. Responses after attempts to induce synaptic plasticity in vehicle-treated animals were thus compared with evoked responses obtained during the assessment of basal synaptic transmission. The same strategy was followed when assessing effects in ligand-treated animals. In addition, plasticity responses were compared in vehicle-treated and ligand-treated animals. Experiments were conducted at intervals of at least 7 days and randomized to avoid any interaction effects.

### Behavior

#### Episodic-Like Spatial Memory Task

An open-field episodic-like memory task (Kart-Teke et al., 2006) was used to test if any components of episodic memory (place—“where” component, temporal order—“when” component, and object identity, or “what” component) are affected by D2R-antagonism. Remoxipride was applied in the same dose as in the electrophysiological experiments (50 μg).

Following icv cannula implantation and a minimum of 10 days of recovery, rats were carefully handled (at least three times on consecutive days for 5 min each) and then habituated in the empty arena (80 × 80 × 80 cm solid gray acrylic box) for three consecutive sessions of 10 min each. In the last habituation session, a sham injection (where the injector tube was simply connected to the guide cannula) was performed 30 min before the animal was placed in the arena. In the interest of keeping the animal numbers to a minimum, each animal was used for this task in two distinct sessions, one for each treatment (vehicle and D2R antagonist). To avoid interference effects, different objects and object locations were used for the two separate sessions. The drug or vehicle treatment sessions occurred in a randomized order for the animals involved.

**Figure 3:**
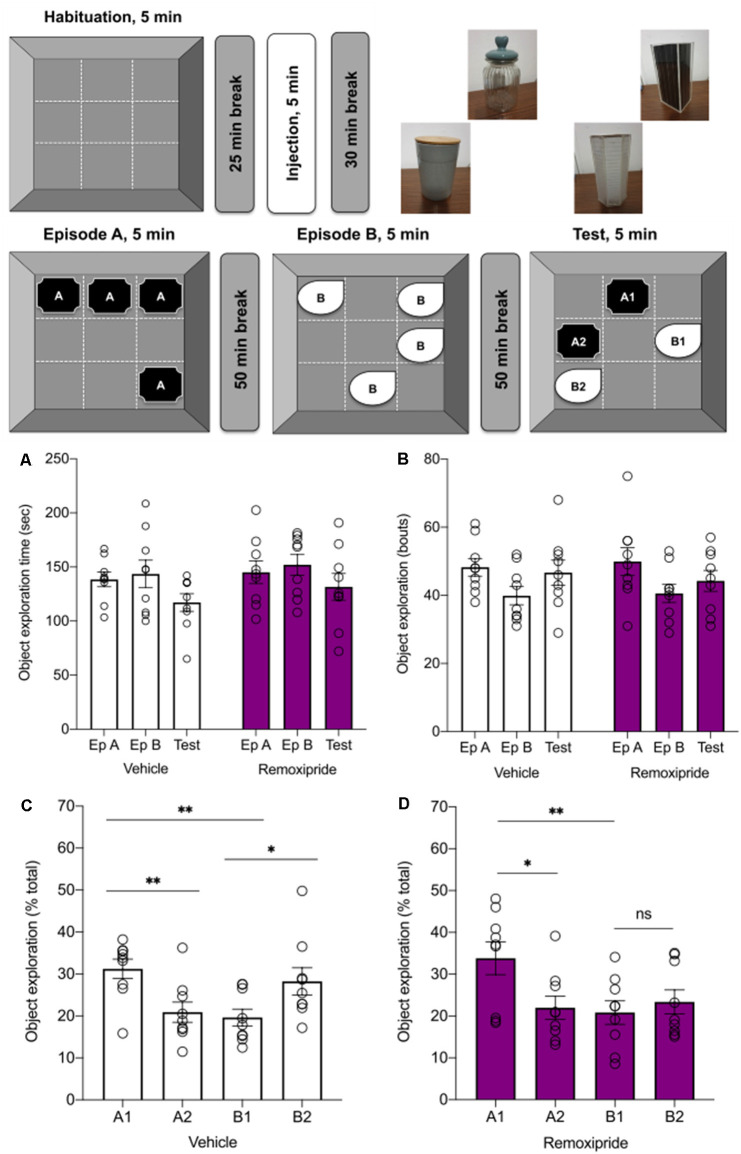
Antagonism ofdopamine D2-like receptors modulates episodic-likememory in rats. Top schema indicates the steps of theepisodic-like memory task. Twenty-five minutes after a5 min habituation phase, animals were treated with vehicle orremoxipride (50 μg). Thirty minutes later Episode A wascommenced. In Episode A (Ep A) animals encountered a novel set ofobjects (“A” objects) in a square arena that was segregated intonine virtual quadrants. Fifty minutes later, during Episode B (Ep B)animals encountered a new set of objects (“B” objects) whereby twoobjects were placed in entirely new quadrants and two objectsoccupied the quadrants of two (previously seen) “A” objects. In theTest phase that occurred 50 min after the conclusion of Ep B, one ofthe “A” objects and one of the “B” objects remained in theirprevious quadrants (A1, B1 “stationary”), and one “A” object (A2)and one “B” object (B2) were moved to previously unoccupied quadrants.The upper right side of the schema shows examples of the objects used. **(A,B)** The object exploration did not vary across treatments, neither with regard to absolute time in seconds (**A**, *n* = 9, *p* = 0.24), nor bouts (**B**, *n* = 9, *p* = 0.99; vehicle: white bars, Remoxipride: filled bars). **(C)** During the test phase, vehicle-treated animals (white bars) preferred the stationary to the displaced objects (*A1* > *A2*, *n* = 9, **p < 0.01) when discriminating between the older familiar objects. By contrast, they explored the displaced in preference to the more recently seen B objects (*B2* > *B1*, *n* = 9, *p < 0.03). They also preferred the stationary older familiar, to the stationary recent familiar, object (*A1* > *B1*, *n* = 9, **p < 0.01). Under remoxipride treatment **(D)**, the rats had similar preferences when discriminating between the familiar older objects (*A1* > *A2*, *n* = 9, *p < 0.05) and stationary objects (*A1* > *B1*, *n* = 9, **p < 0.01), but exhibited no preference for either of the most recently encountered objects (*B2* = *B1*, *n* = 9, ns *p* = 0, 69).

During the training and test trials, no cues were placed on the walls of the arena or fixed on top of them, but distal cues were placed on the surfaces surrounding the arena (approximately, 1 m to 2 m distance from the arena’s floor). The light intensity at the floor level was ca. 6 lux. The floor was divided virtually into nine equally sized quadrants—eight were used as object locations, while the center always remained empty (Figure 3, top panel). Six sets of different objects in quadruplicate were used for the experiments (see examples in Figure 3, right side of top panel). They were made of glass, ceramic material, or hard plastic, and varied in height (20–25 cm), base diameter (7–15 cm), color (white, black, brown, green, light gray, transparent), shape (rectangular or octagonal section, cylinders of various diameters or border shapes), and texture (plain or with various grooved patterns). The weight of each object was such that an adult rat could not displace it. Every set of objects was previously tested in a different group of animals, to ensure that object preference or avoidance effects were not provoked. A video camera was placed at ~2 m above the box floor to record every trial and allow subsequent scoring. The ligand injection was performed 30 min prior to the first training trial in the arena. After each trial, the walls of the arena and the objects were thoroughly cleaned with 70% ethanol, washed with water and dried, to remove any conspecific odors.

Approximately 1 h prior to the start of the experiment, the animals were placed in the room to habituate to the experimental environment. Following that, each rat underwent a 5 min exploration trial in the empty arena and then was placed back in its home cage. The injection was performed 25 min afterwards. Thirty minutes later, the first training trial started. The task consisted of two sample trials and a test trial of 5 min each, interleaved by 50 min of a pause (Figure 3, top panel). At the beginning of a trial, the animal was placed in the center of the arena in a random compass direction. In each of the sample trials, four identical objects were randomly displayed in four out of the eight allocated quadrants of the arena. The object identity was novel for each of the two trials (Figure 3, top panel). The object location from the first trial (Episode A) overlapped for two of the four object locations used in the second sample trial (Episode B), while the other two locations were new. In the test trial, two of the objects used in each of the sample trials were placed according to the following rule: one object from each set (A and B) was placed in a location previously used during its corresponding sample trial—these were identified as objects A1 (“old familiar stationary”) and B1 (“recent familiar stationary”); the other two objects were placed in random locations that had not been not previously occupied in the sample trials by any other object, identified as A2 (“old familiar displaced”) and B2 (“recent familiar displaced”), respectively. Where possible, the training and test sessions were conducted in groups of three rats, allowing interleaved training trials and the counterbalancing of configurations and treatments across animals and sessions.

#### Cumulative Spatial Memory Task

**Figure 4:**
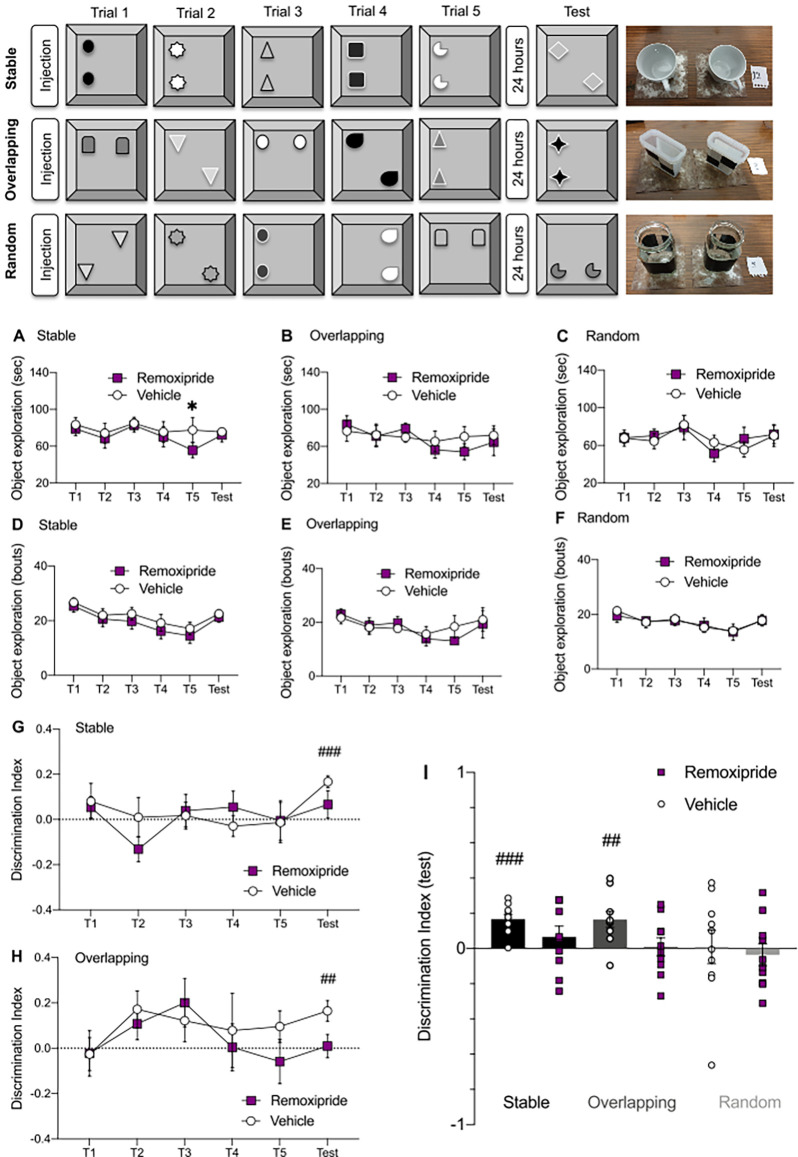
Dopamine D2-like receptor (D2R) antagonism alters the capacity to form cumulative memories. (Top panel) Schematic representation of the Object-Space Task. In all conditions the object identity changed during each trial and in the test session. The right side of the schema shows examples of some of the objects used. In the *stable* condition, the object position remained constant between trials 1–5. Twenty-four hours later, in the test phase, one of the objects was displaced from the familiar location. In the *overlapping* condition, one object remained in a constant position and one object was displaced in Trials 1–5. During the test phase, object identity was changed, but the objects occupied the positions occupied in Trial 5. In the *random* condition object locations varied randomly during all trials and in the test session. **(A)** The total object exploration time (sec) was lower for the last training trial under remoxipride treatment for the *stable* condition (*n* = 10, *p < 0.05). **(B,C)** No differences in object exploration time across trials were found when comparing between vehicle and remoxipride treated animals in either the *overlapping* (**B**, *n* = 10, *p* > 0.05) or (**C**, *n* = 10, *p* > 0.05) *random* conditions. **(D–F)** The number of object exploration bouts did not vary across treatments in any of the three conditions (D—*stable*, E—*overlapping*, F—*random*; *n* = 10, *p* > 0.05). **(G,H)** Comparison of discrimination index (DI) scores across treatments, in each trial, for the *stable* **(G)** and *overlapping* **(H)** conditions. Above chance level DI scores were obtained only in the test trials of the vehicle treated animals both for the *stable* condition (**G**, *n* = 10, ^###^*p* < 0.001) and for the *overlapping* condition (**H**, *n* = 10, ^##^*p* < 0.01). **(I)** DI scores calculated for the test session for every condition and treatment. Black bars—*stable condition*, dark gray bars—*overlapping condition*, light gray bars—*random condition*. Small empty circles represent individual data points for vehicle treatment, while small filled squares reflect individual data points for the antagonist results.

In order to test the effects of the D2R-antagonist on cumulative and/or episodic spatial memory, the Object-Space Task was used (Genzel et al., 2019). Here, the exploration behavior of an individual rat was measured while the animal was sequentially placed in an open field arena where two identical objects were displayed in two of the four corners, following three predefined object-place conditions (Figure 4, top panel).

First, animals underwent an icv cannula implantation (min. 10 days before the first behavioral manipulation) and were handled for at least three individual handling sessions (5 min each), followed by three consecutive days of habituation with the arena (10 min each). The first two habitation sessions were conducted in the empty arena (80 × 80 × 80 cm solid gray acrylic box), while, in the last session, the animals were allowed to explore two identical objects (different than the pairs used for the task) positioned randomly towards the center of the arena. The objects used for the exploration task (4–20 cm height/5–12 cm width, made of metal, glass, or hard plastic) were attached to iron-containing metal plates (12 cm × 12 cm) and magnets were placed under each corner of the arena to support a solid placement of the objects during exploration. At the start of every training session (and also kept during the test session), new bi-dimensional and tri-dimensional cues were placed on the walls, outside of, or above the top of, the arena. A video camera was placed at ~2 m above the box floor to record every trial and allow for subsequent scoring. The light intensity at the floor level was ca. 6 lux. The arena walls and the objects were thoroughly cleaned with 70% ethanol, washed with water and dried, before the beginning of each trial.

An injection was performed 30 min prior to the first training trial (Trial 1), in an empty housing cage. The injection procedure lasted for 5 min and the ligand solution was injected at a speed of 0.5 μl every 30 s. A session consisted of five training trials (5 min each) separated by a 50 min pause, plus a sixth trial of 10 min duration, conducted 24 h after the last training trial, representing the test trial (Figure 4, top panel). Only the initial 5 min of exploration were used for analysis, due to the fact that after this time-point animals stop exploring as the novelty of the situation wears off (Genzel et al., 2019). Two identical items were always included in the arena and the identity of these pairs changed during every trial and during the “test” event. Displaying an entirely different pair of objects for each trial was intended to keep the animal’s exploration time constant, while focusing on the spatial configurations rather than on the object identity (Genzel et al., 2019). Three different training conditions were used so that short-term item-place memory could be discriminated from cumulative semantic like memory:

In the *stable* condition, both item locations remain constant during the five training trials, whereas one of the objects is displaced during the test trial. Here, because the item locations never changed whereas the items’ identity changed from trial to trial, the animals should learn to remember the items’ identity and at the same time learn that the items’ location is constant. Then, on the test day, a new item pair is shown, but in this case, the position of one of the objects is completely novel (Figure 4, top panel). An increase in exploration relative to the other conditions signifies that the animal noticed the novel item location. Thus the *stable* condition, allows robust testing of item-place memory.

In the *overlapping* condition, the item pairs changed on a trial-to-trial basis, one object location remained stable in all training trials, whereas the location of the other object alternated positions from one corner to the other. During the last training event, the stationary object remained in its constant position, and the non-stationary object is moved to the corner that had never been used before (Figure 4, top panel). During the test event, the same object configuration was used as in the last training trial, and a novel pair of matching objects was shown. This condition tested if the animal was capable of forming a cumulative memory by recognizing the least frequently shown configuration of locations of objects, considering all trials.

In the *random* condition (a negative control), the item pair identities changed from trial-to-trial and there was no spatial pattern in the placement of the objects. Furthermore, the item locations during the test were always different than those used in the last training trials. Here, the expectation was that the animals would not manifest any place preference under these conditions.

An animal was usually trained in all three conditions (*overlapping*, *stable*, *random*), for each treatment (once with the vehicle injection and once with the drug), thus allowing within-subject comparisons between the treatments. The training was conducted in groups of four rats (allowing interleaved training trials) and the configurations and order of treatments were counterbalanced among animals and sessions to avoid confounding factors. At least 7 days elapsed between treatments. The experimenter was unaware of the condition or treatment during the subsequent scoring of the animal’s exploration behavior.

### Postmortem Verification of Electrode and Cannula Positions

At the end of experimental procedures, the animals underwent isoflurane anesthesia followed by decapitation and brain extraction. The brain tissue was fixed immediately in 4% paraformaldehyde (PFA) solution (phosphate buffered saline, 0.025 M of PFA, pH of 7.4) for over 1 week and then transferred in 30% sucrose. Thirty micrometer-thick frozen slices were cut on a freezing microtome (Leica Mikrosysteme Vertrieb GmbH, Wetzlar, Germany), mounted on glass slides, air-dried, and stained in 0.1% cresyl violet (Hansen and Manahan-Vaughan, 2015). The sections were examined using a light microscope (Leica Mikrosysteme Vertrieb GmbH, Wetzlar, Germany) and photomicrographs were captured *via* a digital video camera system (HD camera, Microsoft). Where incorrectly implanted electrodes or cannulas were detected, the whole data set for that particular animal were removed from the results.

### Data Analysis

The raw data (electrophysiological data, or Solomon Coder scoring values for behavior) were visualized and analyzed statistically with Prism 9 software (GraphPad; RRID:SCR_002798).

For electrophysiological recordings, the raw values (fEPSP slope values determined from the average of five consecutive responses, evoked at 0.025 Hz for each time-point) were collected. Data were then expressed as the mean percentage ± standard error of the mean (SEM), calculated from the average of the first six baseline time-points. To identify differences between baseline conditions and experimental ones, a two-way factorial analysis of variance with repeated measures (rmANOVA) was used, followed by a Fisher’s Least Significant Difference (LSD) test, to further explore and compare between means of the groups. The significance level was set at a *p*-value < 0.05. Where rmANOVA revealed a significant difference, interaction effects at the level of “time” and “treatment group” were assessed.

For behavioral experiments, the time spent exploring the objects(measured in seconds) and the total count of visits of each object during each trial (bouts) were scored offline with the Solomon Coder software^1^ (RRID: SCR_016041). For both tasks, exploration was deemed to have occurred when the animal’s snout and/or forepaws engaged in direct contact with the object. For the episodic-like memory task, the object preference during the test session was calculated as the mean ± SEM for the percentage of exploration time (when compared to 100% as determined by the total object exploration during the test session). For both tasks, the experimenter was unaware of the animal’s treatment or condition whilst scoring the exploratory behavior.

For the Object Space Task, the Discrimination Index (DI) was calculated as the difference in exploration time between the novel and stable locations, divided by the total exploration time. A score closer to −1 signified preference for stable location; a score closer to +1 signified preference for the less stable location, while a score of zero meant no preference for object location. For the episodic-like memory task, a one-way ANOVA was used to determine object exploration variability across the four objects displayed during the test session, followed by multiple comparisons (*t*-tests for pairs of objects) and the Fisher’s LSD test. A two-way ANOVA was also used to probe for significant differences in exploration time or bouts, for each trial, across treatments. For the Object Space Task, the differences in object exploration time or bouts and discrimination index scores across treatments were assessed with multiple paired *t*-tests with no correction. In order to test for the presence of memory in each of the conditions and treatments, a one-sample *t*-test (compared to chance levels) was applied to the DI calculated for each trial.

## Results

The current study aimed to assess the involvement of D2R on hippocampal synaptic plasticity in the CA1 region and spatial memory. To do so, we tested the effects of the D2-like receptor antagonist remoxipride, at a dose of 50 μg that does not affect basal synaptic transmission in the dentate gyrus (Manahan-Vaughan and Kulla, 2003) or CA3 region (Hagena and Manahan-Vaughan, 2016). Synaptic plasticity was assessed in freely behaving rats and the impact of D2R on synaptic plasticity in the hippocampal CA1 region and on episodic-like and semantic-like memory was investigated.

### Pharmacological Antagonism of Dopamine D2R Does Not Alter Basal Synaptic Transmission at SC-CA1 Synapses *In vivo*

We first clarified whether the chosen antagonist dose affects basal synaptic transmission in the CA1 region of freely behaving rats. When we applied remoxipride (50 μg) and subsequently recorded fEPSPs for a 4 h period, we detected no significant differences in evoked responses compared to vehicle-treated animals (Figures 1A,D, ANOVA: *F*_1, 12_ = 0.77, *p* = 0.40; interaction effect: *F*_23, 276_ = 0.57, *p* = 0.94, *n* = 7). Therefore, we used this ligand dose to investigate the effect of D2R-antagonism on CA1 synaptic plasticity.

### Pharmacological Antagonism of Dopamine D2R Prevents Initiation of STP and LTP at SC-CA1 Synapses *In vivo*

To investigate whether D2R contribute to CA1 synaptic potentiation, we selected two afferent stimulation protocols: one induces short-term potentiation (STP; weak high frequency stimulation: wHFS, comprising 100 Hz, three trains of 10 pulses each, applied at 10 s interval; Manahan-Vaughan and Reymann, 1995a; Manahan-Vaughan, 1997; Manahan-Vaughan et al., 1999), while the other protocol induces long-term potentiation (LTP; high frequency stimulation: HFS, comprising 100 Hz, four trains of 30 pulses each, applied at 5 min interval; Kemp and Manahan-Vaughan, 2004; Lemon and Manahan-Vaughan, 2006).

In vehicle-treated controls, wHFS led to STP that lasted less than 30 min, compared to responses evoked in vehicle-treated test-pulse stimulated controls (not shown, ANOVA: *F*_1, 14_ = 7.10, *p* < 0.05; interaction effect: *F*_3, 42_ = 4.46, *p* < 0.01, *n* = 8). When wHFS was applied in the presence of remoxipride (50 μg), STP was significantly prevented, compared to vehicle-treated controls (Figures 1B,E, ANOVA: *F*_1, 12_ = 8.11, *p* < 0.05; interaction effect: *F*_2, 24_ = 6.32, *p* < 0.01, *n* = 7).

When we applied HFS to vehicle-treated controls, LTP was expressed that lasted over 4 h (Figures 1C,F). Effects were significantly different when compared to vehicle-treated test-pulse stimulated controls (not shown, ANOVA: *F*_1, 20_ = 63.27, *p* < 0.001; interaction effect: *F*_17, 340_ = 4.30, *p* < 0.001, *n* = 11). HFS in the presence of remoxipride (50 μg) fully prevented LTP, including the early phases of potentiation (Figures 1C,F, ANOVA: *F*_1, 10_ = 13.69, *p* < 0.01; interaction effect: *F*_17, 170_ = 1.57, *p* = 0.08, *n* = 6).

These results indicate that both early and late phases of synaptic potentiation are prevented by antagonism of D2R.

### Short-Term Depression at SC-CA1 Synapses Is Prevented by Dopamine D2R Antagonism

To establish whether the early phase of synaptic depression is also affected by D2R, we used a low-frequency stimulation (LFS) protocol (1,800 paired pulses/900 pairs, applied at 1 Hz with a 25 ms inter-pulse interval) to induce short-term depression (STD). This protocol, when applied to vehicle-treated animals, resulted in STD of SC-CA1 synapses that lasted for less than an hour (Figures 2A,B). Effects were significantly different from responses evoked in vehicle-treated test-pulse stimulated controls (not shown, ANOVA: *F*_1, 16_ = 9.83, *p* < 0.01; interaction effect: *F*_5, 80_ = 6.79, *p* < 0.001, *n* = 9). When LFS was applied in the presence of remoxipride (50 μg), STD was completely prevented (Figures 2A,B, ANOVA: *F*_1, 10_ = 5.71, *p* < 0.05; interaction effect: *F*_5, 50_ = 1.48, *p* = 0.21, *n* = 6).

These results indicate that D2R bidirectionally regulate synaptic plasticity in the CA1 region with effects specifically targeting the early phase of both synaptic potentiation and synaptic depression.

### D2R Antagonism Mildly Alters the “Where” Component of Short-Term Episodic-Like Memory in Rats

In order to assess whether D2R contribute to hippocampal episodic-like learning and memory, we selected a behavioral task that disambiguates aspects of “what”, “where”, and “when” features of episodic-like memory in rats (Kart-Teke et al., 2006).

With regard to general object exploration, no differences were found across treatments in terms of the total object exploration time (Figure 3A, ns *p* = 0.24, *n* = 9) or the number of exploration bouts (Figure 3B; ns *p* = 0.99, *n* = 9) leading to the conclusion that the antagonist did not directly alter the animals’ exploration capacity or interest in exploring the objects.

When the animals’ episodic-like memory was tested, it was found that a specific object preference hierarchy emerged in vehicle-treated animals. Here, a one-way ANOVA revealed significant differences in exploration across the four objects (Figure 3C, One-way ANOVA: *F*_3, 32_ = 4.92, **p < 0.01, *n* = 9). With regard to the stationary object condition, the animals preferred the “old familiar” (A1) stationary object over the “recent familiar” stationary one (B1; Figure 3C, one-way ANOVA, Fisher’s LSD *t*-test: *t*_32_ = 3.23, **p < 0.01, *n* = 9) suggesting that the rats discriminated both the identity of the object (“what”) and the order of their presentation (“when”). If, instead, we compared exploration of the two pairs of objects (A1 vs. A2, or B1 vs. B2), the rats explored the stationary “old familiar” object (A1) for longer compared to the displaced “old familiar” object (A2; Figure 3C, one-way ANOVA, Fisher’s LSD *t*-test: *t*_32_ = 2.88, **p < 0.01, *n* = 9). By contrast, with regard to the most recently displayed B objects, animals preferred to explore the displaced object (B2) more than the stationary one (B1; Figure 3C, one-way ANOVA, Fisher’s LSD *t*-test: *t*_32_ = 2.40, *p < 0.03, *n* = 9).

These findings are consistent with those of Kart-Teke et al. (2006); and point to an interaction between the recency (“when”) and spatial displacement (“where”) factors in episodic-like memory formation.

Summing up these results, the control animals’ object preference comprises: A1 > B1 (“what” and “when”—animals preferred the object seen more distantly in the past when comparing between the stationary objects from each pair); A1 > A2 (“when” over “where”—when objects shown most distantly in the past are considered, then the stationary object is preferred over the displaced object); and B1 < B2 (“where”—when objects shown most recently in the past are considered, displaced objects are preferred over the stationary ones). Considering the total exploration testing the test session, the following relationship is evident: A1 > B2 > B1 = A2.

Under remoxipride (50 μg) treatment, one-way ANOVA revealed that the animals still discriminated between the objects during the test session (Figure 3D, ANOVA: *F*_3, 32_ = 4.54, **p < 0.01, *n* = 9). The A1 > B1 discrimination was unaltered (Figure 3D, one-way ANOVA, Fisher’s LSD *t*-test: *t*_32_ = 3.31, **p < 0.01; *n* = 9). Similarly, the animals preferred object A1 over A2 (A1 > A2), although the discrimination was less strong in the presence of remoxipride (Figure 3C, one-way ANOVA, Fisher’s LSD *t*-test:* t*_32_ = 2.69, *p < 0.05, *n* = 9). However, when exploration of the most recently displayed objects was compared (B1 vs. B2; and in contrast to results obtained in vehicle-treated animals), no significant discrimination was found in the presence of remoxipride (B1 = B2; Figure 3D, one-way ANOVA, Fisher’s LSD *t*-test: *t*_32_ = 0.41, ns *p* = 0.69; *n* = 9). These results indicate that D2R antagonism impaired the retention of the “where” component of the episodic-like memory task, leaving the “what” (item identity) and “when” (temporal) components intact (Figure 3D, A1 > B2 = B1 = A2).

When, however, performance in vehicle-treated rats was compared with performance in antagonist-treated rats, no significant effects were detected (two-way rmANOVA: *F*_1, 32_ = 6.37, *p* = 0.9998, each *n* = 9). This suggests that D2-like receptors only mildly contribute to “what-where-when” memory, with effects appearing only within the remoxipride treated group and occurring with regard to recent object-place.

### D2R Contribute to Long-Term Cumulative Memory Formation

Given the abovementioned finding that the spatial component of an episodic-like task was mildly affected by D2R antagonism, we went on to assess the involvement of D2R in semantic-like spatial memory, based on knowledge accumulation over multiple episodes (Genzel et al., 2019). The task tests memory based on the natural tendency of rodents to preferentially explore novel items.

The total object exploration time was measured for every trial and compared across treatments for each condition. In the *stable* condition, the objects’ locations remained constant across all training trials, and only changed during the “test” whereupon one item location was changed. The identities of both objects changed on a trial-to-trial basis, meaning that over time the animal learned that the item location was negligible but the item identity changed on a daily basis. The consistent object exploration during the training trials, despite the fact that the items’ positions did not change, reflects the fact that interest in the novel objects was sustained across trials. An interference by the antagonist of acute object recognition memory can be excluded, based on the results of the episodic-like memory task, where we found that remoxipride did not alter object identity memory (Figure 3D; A1 vs B1 discrimination). Interestingly, in the *stable* condition of the cumulative memory task, remoxipride-treated animals exhibited a lower object exploration time in the last training trial (Trial 5), when they were compared to vehicle-treated controls (Figure 4A, Multiple paired *t*-test: *t*_9_ = 2.27, ^*^p < 0.05, *n* = 10), although no difference in the number of exploration bouts was evident in that particular trial (Figure 4D, Multiple paired *t*-test: *t*_9_ = 1.79, *p* = 0.11, *n* = 10).

The *overlapping* condition tested if the animal recognized the least frequently shown configuration of object location (cumulative memory), and the *random* condition served as a negative control (no place preference should occur). For both the *overlapping* and *random* conditions, no differences were found in either the total object exploration time or the number of exploration bouts for any of the trials (Figures 4B,C,E,F; Multiple paired *t*-test: *p* > 0.05, *n* = 10). The fact that the D2R antagonist slightly altered the exploration only in the *stable* condition and only for the last training trial, where the degree of novelty was lower compared to the other conditions and trials of the task, might be related to dopaminergic dependency of novelty detection (Kempadoo et al., 2016; Duszkiewicz et al., 2019).

Next, we sought to find if the animals managed to discriminate the most novel object location in the test sessions, by looking at the discrimination index (DI). We compared each treatment and condition results to chance levels (statistical results marked with hashtags in Figure 4).

In the *stable* condition, only the test session resulted in a DI above chance levels in vehicle-treated animals (Figures 4G,I), as might be expected given the findings of others (Genzel et al., 2019; Figure 4I, black bar with small circles; *t*_9_ = 6.32, ^###^*p* < 0.001, *n* = 10). By contrast, following remoxipride treatment (50 μg), animals did not reach the significance threshold in discriminating the novel location during the test session, when compared to chance levels (*t*_9_ = 1.09, ns *p* = 0.30, *n* = 10).

In the *overlapping* condition, the vehicle-treated animals discriminated the most novel location during the test session (Figures 4H,I: *t*_9_ = 3.64, ^##^*p* < 0.01, *n* = 10). Interestingly the antagonist significantly altered memory performance in the *overlapping* test session, lowering it to below chance levels (paired *t*-test: ns *p* > 0.05; *n* = 10).

Finally, in the *random* condition, where the animals were expected not to manifest any place preference, the DI did not vary from chance levels for any trial (Figure 4I).

Taken together, these results indicate that D2R antagonism impaired the animals’ capacity to discriminate between the most and least novel object locations both when the cumulative learning was simpler (in the *stable* condition, when they were exposed for five consecutive times to the same “old” configuration before presenting the novel location during test session), but also in the more complex *overlapping* condition, where the animals needed to extract the fixed (*overlapping*) location rule across the trials and then be able to identify it 24 h later.

## Discussion

ic plasticity in the dorsal hippocampal CA1 region and on episodic-like and semantic-like spatial memory in rats. Our *in vivo* electrophysiology experiments showed that the initiation and maintenance of both synaptic potentiation and depression are prevented by dopamine D2R antagonism, at a dose that does not alter basal synaptic transmission. Moreover, the antagonist, when applied before encoding at the same dose, alters both short-term episodic-like memory and long-term semantic-like memory.

### Dopamine D2R Bidirectionally Modulate Schaffer Collaterals-CA1 Synaptic Plasticity

The D1-like family of dopamine receptors plays an important role in supporting the late maintenance phases of both hippocampal LTP and LTD (Navakkode et al., 2007; Hansen and Manahan-Vaughan, 2014; Wiescholleck and Manahan-Vaughan, 2014; Hagena et al., 2016). By contrast, our current results indicate that D2R contribute to the *early* induction phases of LTP and LTD. This finding, in freely behaving rats, contrasts with results obtained using the hippocampal slice preparation. For example, Frey and colleagues (Frey et al., 1990) reported that D2R antagonism only alters late-LTP and has no effect on LTP induction in rat hippocampal slices. Another *in vitro* study reported that genetic deletion of D2R prevents LTP and LTD but leaves the very early phases intact (Rocchetti et al., 2015). Effects are emulated by a D2/D3R antagonist (Rocchetti et al., 2015). These differences from our own results may derive from the fact that dopamine afferents are severed in the slice preparation and thus, intrinsic DA tonus is absent. In line with this, studies conducted in mice *in vivo* that either lacked D2R or in which CA1 D2R were silenced by siRNAs, reported that D2R are necessary for both the induction and maintenance of LTP in CA1 synapses (Espadas et al., 2021).

We have recently reported that highly localized gene encoding occurs in the hippocampus during the strengthening of synaptic plasticity by spatial learning (Hoang et al., 2021). By contrast, strong afferent protocols that induce highly robust forms of LTP and LTD result in generalized and indiscriminate immediate early gene encoding across the hippocampus (Hoang et al., 2021). In this context, it is interesting to note that slow-onset potentiation that is induced by learning activity is impaired in mice that either lack D2R, or undergo local silencing of the receptor in the CA1 region (Espadas et al., 2021). Taken together, this suggests that stronger LTP and LTD protocols (used *in vitro*) may mask a regulation by D2R of early LTP that becomes apparent using milder plasticity protocols *in vivo*, or is evident in studies where synaptic plasticity was facilitated by learning. In line with this, a role for D2R in spike-timing-dependent plasticity in hippocampal slices was recently reported (Cepeda-Prado et al., 2021).

Although it cannot be entirely excluded, it does not seem likely that the effects on synaptic plasticity we report here can be explained by D2R-mediated regulation of presynaptic glutamate release probability at CA1 synapses. For example, stimulus-response relationships and paired-pulse facilitation were reported to be unchanged in the hippocampus of transgenic mice that lack D2R and in D2R-silenced Drd2-siRNA mice (Espadas et al., 2021). Furthermore, we previously reported that a dose-dependent *decrease* in basal synaptic transmission occurs in the hippocampus of freely behaving rats when a D2R agonist is applied (Manahan-Vaughan and Kulla, 2003). Thus, the modulation of excitability levels driven by D2R activation seems rather to go in the direction of depression rather than excitation, supporting the idea that the modulation of synaptic plasticity is not driven “merely” by improvements in excitation levels, especially given that in the present study we observed an inhibition of *both* LTP and synaptic depression by D2R antagonism. A similar bidirectionality of effects has been reported in the hippocampal slice preparation (Rocchetti et al., 2015). Another study, conducted both *in vivo* and using whole-cell CA1 cells recordings, reported that D2R antagonism inhibited, while a D2R agonist mimicked, the LFS-mediated depotentiation effects in a kindling rat model (Sadeghian et al., 2020), pointing to a role of these receptors in depotentiation, a plasticity process that is biochemically distinct to both LTP and LTD (Lee et al., 1998; Kulla et al., 1999). This raises the question as to how the receptor can mediate such opposing effects on synaptic efficacy.

Short-term depression was prevented by D2R antagonism in the present study. This finding aligns with the fact that D2R are negatively coupled to adenylyl cyclase (AC; Beaulieu and Gainetdinov, 2011), and the activation of G-protein-coupled receptors that are negatively coupled to AC is required for induction of LTD (Santschi et al., 2006). Furthermore, D2R antagonism (with sulpiride) prevents LTD *in vitro* (Rocchetti et al., 2015). However, others have reported that D2R antagonism in CA1 slices (with LY-171555) *amplifies* LTD (Chen et al., 1996). A possible explanation for these rather conflicting results is that the ligands used in the different studies may bind to both D2 and D3 receptors. This may be the case for sulpiride that, in autoradiographical studies, was reported to bind to both receptors (Landwehrmeyer et al., 1993). Furthermore, LY-171555 (quinpirole) has been reported to preferentially activate D3-receptors in rats (Collins et al., 2005). By contrast, remoxipride (used in the present study) exhibits a high binding specificity for D2-receptors (Farde and Bahr, 1990). Thus, as yet, a clear picture has not emerged as to the possible role of D2R in synaptic depression.

D2R occur in short (D2S, presynaptic) or long (D2L, postsynaptic) isoforms (Picetti et al., 1997; Usiello et al., 2000). The antagonist we used, remoxipride (Ögren et al., 1984), exhibits a higher affinity for the D2S isoform (Malmberg et al., 1993), whereas haloperidol, has a high affinity for the D2L isoform (Usiello et al., 2000), and both quinpirole and sulpiride bind to both isoforms (Itokawa et al., 1996; Kuzhikandathil et al., 1998; Centonze et al., 2004). The two D2R isoforms play different roles in synaptic plasticity modulation: The D2S isoform is mostly expressed presynaptically, regulates synaptogenesis, and gates dopamine release by means of its autoreceptor function (Congar et al., 2002; Fasano et al., 2008; Ford, 2014), whereas the D2L isoform is predominantly localized postsynaptically (Khan et al., 1998). Interestingly, in animals lacking D2L, activation of the D2S isoform inhibits D1R-mediated functions (Usiello et al., 2000), pointing to a signaling interference between different dopamine receptors. In this respect, Lee and colleagues (Lee et al., 2004) identified a calcium signaling pathway that depends on D1-D2 receptor co-activation, which cannot be activated by either receptor alone and might involve Gq coupling. Therefore, the fact that the induction of all forms of synaptic plasticity tested in our study was blocked by an antagonist that preferentially binds to D2S isoforms suggests that a presynaptic mechanism underlies the effects seen. Thus, the antagonist may have altered presynaptic (auto-receptor) regulation of dopamine release. The effect can be expected to enhance dopamine release. One could speculate that this might trigger over-activation of D1-like receptors (Usiello et al., 2000) and a decrease in DA transporter action (Mayfield and Zahniser, 2001): another consequence of D2S receptor antagonism may be the hindrance of D1-D2 receptor calcium-signaling (Lee et al., 2004) necessary for effective plasticity induction.

The bidirectional modulation of CA1 synaptic plasticity by D2R may also be supported by their influence on N-methyl-D-aspartate receptors (NMDAR). For example, it was shown that D2R agonism decreases the surface expression of GluN1 NMDAR subunits in cultured neurons, whereas D2R antagonism prevents this effect (Gao and Wolf, 2008). D2R activation reduces spine number in DA neurons which also co-release glutamate (Fasano et al., 2008; Jia et al., 2013; Iino et al., 2020). This occurs by means of a mechanism that acts *via* GluN2B NMDAR subunits and is also cAMP-dependent (Jia et al., 2013). Given that the bidirectionality of NMDAR-dependent synaptic plasticity may especially rely on the GluN2B subunit (Shipton and Paulsen, 2013), and this subunit is also important in the maintenance of synaptic potentiation (Ballesteros et al., 2016), these inter-relationships of D2R and NMDAR may explain the D2R blockade of synaptic plasticity induction.

### The Encoding of Both Episodic-Like and Semantic-Like Memories Involves Dopamine D2R

In our current study, we observed a specific regulation by D2R of spatial components of episodic-like and semantic-like memory. These findings are consistent with studies conducted on human subjects. For example, various studies indicated that an optimal balance between D2 receptors and dopamine availability is needed to achieve effective episodic and working memory performance (Aalto et al., 2005; Takahashi et al., 2008; Papenberg et al., 2020). Hippocampal D2R are important for hippocampus-specific functions such as memory consolidation, as well as for facilitating interactions with other areas, such as the prefrontal cortex (modulated by D1Rs) or striatal areas, processes which all subserve the acquisition of episodic memory (Takahashi et al., 2008; Nyberg et al., 2016).

In rodent studies, it was shown that a rat‘s ability to acquire spatial reference memory in a water maze depends on the integrity of mesohippocampal dopaminergic connections (Gasbarri et al., 1996). In line with this observation, D2R antagonism prevents the cognitive enhancement effects of angiotensin in a conditioned avoidance response paradigm and in an object recognition task (Braszko, 2006). D2R antagonism also prevents the acquisition of spatial avoidance behavior in a carousel maze (Prokopova et al., 2012), as well as novel object discrimination (Lee and Chirwa, 2015). Moreover, D2R antagonism prevents the renewal of a previously learned experience following extinction learning, suggesting that receptor antagonism may promote the disambiguation of similar spatial experiences (André and Manahan-Vaughan, 2016).

In both our behavioral tasks, we applied the antagonist prior to encoding (i.e., prior to Trial 1) in order to test its effects on early (plasticity-dependent) learning processes (Kemp and Manahan-Vaughan, 2004; Hoang et al., 2021). The episodic-like and semantic-like memory tasks were based on the animals’ innate preference for novelty. This allowed us to assess the effects of altering dopamine–mediated novelty detection under D2R blockade conditions. This specific aim was motivated by the fact that the CA1 region is thought to play an important role in novelty detection in the VTA-hippocampal loop (Manahan-Vaughan and Braunewell, 1999; Kemp and Manahan-Vaughan, 2004; Lisman and Grace, 2005; Tsetsenis et al., 2021), a process that is strongly dependent on dopaminergic signaling, which also known for its role in novelty detection processes (Clos et al., 2019; Duszkiewicz et al., 2019). Our results in the episodic-like task and the cumulative semantic-like paradigm both showed that the rats performed worse in recognizing novel features of the objects in the presence of the D2R antagonist, compared to controls, during the final test sessions. These findings in rats align with reports from behavioral studies in mice in which D2R were either knocked-out or silenced specifically in the CA1 region, which demonstrated that spatial working memory (Glickstein et al., 2002), spatial and recognition learning and memory (Rocchetti et al., 2015), reversal learning and even associative learning, such as in trace eyeblink conditioning (Espadas et al., 2021) are all impaired when D2R function is disrupted. Others have reported that D2R-antagonism can affect movement initiation and execution (Amalric et al., 1993; Hauber, 1996; Parr-Brownlie and Hyland, 2005). Side-effects such as these must be taken into account in any memory task that requires that an animal moves through space. Close monitoring of the animals’ movements in the present study did not reveal any changes. Here, the environments used were moderate in size (80 × 80 cm) and did not require extensive locomotion. Both animal cohorts easily reared onto the objects used in the episodic memory like task (20–25 cm high), and neither exploration time nor number of explorations bouts varied across treatments in the behavioral studies. This suggests that effects on motor behavior at the dose of remoxipride used in the present study were negligible.

In the episodic-like memory task, our animals successfully discriminated between old and new familiar objects (A1 > B1), as well as between old displaced and old stationary objects A1 > A2), but could not discriminate between the most recently displaced objects in the final test session (B1 = B2) under remoxipride treatment. One potential explanation for this effect could be that the antagonist allowed first memories to form (A objects), but disrupted the discriminated encoding of the second set of objects (B objects). Novel item encoding is supported by structures such as the perirhinal cortex (Sethumadhavan et al., 2020). Although this cortex expresses D2R (Goldsmith and Joyce, 1994), it is unclear as yet whether D2R antagonism affects object memory acquisition in this structure. The fact that initial object memory appears to be spared, whereas information updating about the spatial location of the objects is impaired, suggests that D2R may exert specific effects on spatial, rather than on object, memory. We explored this possibility in the cumulative object-space task (Genzel et al., 2019). Here, D2R antagonism impaired memory of the simpler (*stable*) and the more complex (*overlapping*) spatial memory tasks. As such, the animals failed to form cumulative spatial memories. This is consistent with an impairment, by D2R antagonism, of the acquisition of spatial novelty and the corresponding updating of spatial representations.

### Final Remarks

In conclusion, our study shows that D2R receptors bidirectionally regulate synaptic plasticity in the hippocampal CA1 region of freely behaving rats. Effects specifically target the early phase of synaptic plasticity, consistent with an autoreceptor-mediated regulation of dopamine release (Congar et al., 2002), or an alteration by D2R of NMDAR function (Gao and Wolf, 2008). Furthermore, effects on learning behavior are tightly associated with the spatial components of both episodic-like and semantic-like memory, consistent with recent reports that D2R antagonism impairs spatial cognition in human subjects (Naef et al., 2017). Finally, given the specificity of remoxipride for the D2S isoform of D2R, our findings suggest that this isoform may be particularly important for the regulation of these processes.

## Data Availability Statement

The raw data supporting the conclusions of this article will be made available by the authors, upon reasonable request.

## Ethics Statement

The animal study was reviewed and approved by Ethics committee of the federal state of North Rhine Westphalia (NRW; Landesamt für Naturschutz, Umweltschultz und Verbraucherschutz, NRW, Bezirksamt Arnsberg).

## Author Contributions

The study was designed by DM-V and V-MC. Experiments were conducted by V-MC and analyzed by V-MC and DM-V. Both authors wrote the article. Both authors approved the submitted version.

## Funding

This work was supported by a grant from the German Research Foundation (Deutsche Forschungsgemeinschaft, DFG) to DM-V (SFB 874/B10, project number: 122679504).

## Conflict of Interest

The authors declare that the research was conducted in the absence of any commercial or financial relationships that could be construed as a potential conflict of interest.

## Publisher’s Note

All claims expressed in this article are solely those of the authors and do not necessarily represent those of their affiliated organizations, or those of the publisher, the editors and the reviewers. Any product that may be evaluated in this article, or claim that may be made by its manufacturer, is not guaranteed or endorsed by the publisher.

## Footnotes

^1^ https://solomon.andraspeter.com/
